# Factors Determining Survival and Retention among HIV-Infected Children and Adolescents in a Community Home-Based Care and a Facility-Based Family-Centred Approach in Kampala, Uganda: A Cohort Study

**DOI:** 10.1155/2014/852489

**Published:** 2014-04-01

**Authors:** W. Massavon, L. Barlow-Mosha, L. Mugenyi, W. McFarland, G. Gray, R. Lundin, P. Costenaro, M. M. Nannyonga, M. Penazzato, D. Bagenda, C. P. Namisi, D. Wabwire, M. Mubiru, S. Kironde, D. Bilardi, A. Mazza, M. G. Fowler, P. Musoke, C. Giaquinto

**Affiliations:** ^1^Department of Paediatrics, University of Padua, Via Giustiniani 3, 35128 Padua, Italy; ^2^St. Raphael of St. Francis Hospital (Nsambya Hospital), Kampala, Uganda; ^3^Makerere University, Johns Hopkins University Research Collaboration, Kampala, Uganda; ^4^Infectious Diseases Research Collaboration, Mulago Hospital Complex, Kampala, Uganda; ^5^Department of Global Health Sciences, University of California San Francisco, 50 Beale Street, 12th Floor, San Francisco, CA 94105, USA; ^6^University of Witwatersrand, 1 Jan Smuts Avenue, Braamfontein 2000, Johannesburg, South Africa; ^7^Department of Global Heath and Population, Harvard University School of Public Health, Boston, MA, USA; ^8^Santa Chiara Hospital, Via Largo Gold Medals 9, 38122 Trento, Italy; ^9^Department of Pathology, Johns Hopkins School of Medicine 600 N. Wolfe Street/Carnegie 43 Baltimore, MD 21287, USA; ^10^Department of Paediatrics and Child Health, Makerere University, Kampala, Uganda

## Abstract

We describe factors determining retention and survival among HIV-infected children and adolescents engaged in two health care delivery models in Kampala, Uganda: one is a community home-based care (CHBC) and the other is a facility-based family-centred approach (FBFCA). This retrospective cohort study reviewed records from children aged from 0 to 18 years engaged in the two models from 2003 to 2010 focussing on retention/loss to follow-up, mortality, use of antiretroviral therapy (ART), and clinical characteristics. Kaplan Meier survival curves with log rank tests were used to describe and compare retention and survival. Overall, 1,623 children were included, 90.0% (1460/1623) from the CHBC. Children completed an average of 4.2 years of follow-up (maximum 7.7 years). Median age was 53 (IQR: 11–109) months at enrolment. In the CHBC, retention differed significantly between patients on ART and those not (log-rank test, adjusted, *P* < 0.001). Comparing ART patients in both models, there was no significant difference in long-term survival (log-rank test, *P* = 0.308, adjusted, *P* = 0.489), while retention was higher in the CHBC: 94.8% versus 84.7% in the FBFCA (log-rank test, *P* < 0.001, adjusted *P* = 0.006). Irrespective of model of care, children receiving ART had better retention in care and survival.

## 1. Background

Sub-Saharan Africa (SSA) is home to the vast majority of infants, children, and adolescents living with HIV and morbidity and mortality remain high [1–3]. For example, mortality among HIV-infected children has been measured at 4.3% per year in East Africa and 8.3% in West Africa [4, 5]. A recent meta-analysis conducted in SSA reported a higher risk of early death among perinatally infected children [6]. Studies have also shown that substantial proportions of children and adolescents initiate treatment in SSA with advanced disease (46.3%–72.0%) and comorbidities such as tuberculosis (TB) (5.7%–34.0%) and malnutrition (33%–54%) that tend to be associated with early mortality and poor clinical outcomes [7–10].

Significant child mortality can be averted if antiretroviral therapy (ART) is started early [11–14]. However, despite overwhelming evidence demonstrating the benefits of ART, in practice high mortality and poor retention persist among HIV-infected children and adolescents in care in the resource-limited settings of SSA. In addition to scarce resources for programmes for children, the situation is compounded by a combination of factors including late HIV diagnosis, missed opportunities to initiate ART, health care programmes not tailored to the needs of the infected child and their family, and logistic bottlenecks in implementation of care and treatment programmes [15, 16]. Initiation of ART even among children known to be eligible may be missed. For instance, in a study of ART-eligible children in The Gambia, only 32.7% started ART, 47.1% were lost to follow-up, and 13.5% died before initiating ART [17].

Retention in care while awaiting ART eligibility can also be a challenge. As an illustration, retention varied from 71% to 95% and 62% to 93% at 12 and 24 months, respectively, among children and adolescents in ART programmes in several countries of SSA [18]. A prior study in Uganda showed that even with frequent CD4 monitoring, HIV-infected children experienced significant clinical events while ineligible for ART according to the 2006 WHO guidelines [19]. Another study in Uganda showed that mortality was highest among HIV-infected children under two years [20]. Given this situation, it is important to assess factors that determine survival and retention in care among HIV-infected children and adolescents in care in resource-limited settings.

The present study focuses on retention and survival in two different ART delivery models for HIV-infected children and adolescents in Kampala, Uganda. One is a facility-based, family-centred approach (FBFCA) adopted by the mother to child transmission (MTCT)-Plus programme of the Makerere University-Johns Hopkins University (MU-JHU) Research Collaboration. The other is a community home-based care (CHBC) implemented by the children's HIV programme of the Home Care Department of Nsambya Hospital. The American Academy of Paediatrics defined family-centred care as based on the understanding that the family is the child's primary source of strength and support [21]. Beyond that, the family provides an enabling environment for using index HIV patients to reach other infected and affected family members, build family support for therapy and chronic care, integrate other medical needs for the family, and thus enhance uptake of services for HIV and other medical conditions for the family as a unit [22]. In general, CHBC includes any form of care (physical, psychosocial, palliative, and spiritual) given to the sick and the affected in their own homes and care extended from the hospital or health facility to their homes through family participation and community involvement [23, 24].

Factors that determine retention and survival of HIV-infected children and adolescents in these health care models are not well understood. The present study therefore aims at identifying factors that determine these outcomes for the CHBC of Nsambya Hospital and the FBFCA of MU-JHU. We also examine pre-ART deaths among children and adolescents in the CHBC to compare mortality rates prior to and after ART initiation and to ascertain whether children experiencing mortality prior to initiating ART had met the 2010 WHO [25] or the 2011 updated United States (US) [26] treatment guidelines for initiating ART or not.

## 2. Methods

### 2.1. Study Design, Setting, and Population

This retrospective cohort study covered eight years of records review (2003 to 2010) from two facilities implementing HIV paediatric programmes in Kampala, Uganda. They included the children's HIV programme of the Home Care Department of Nsambya Hospital, which uses community home-based care (CHBC) and the MTCT-plus programme of MU-JHU Research Collaboration, which adopts a facility-based family-centred approach (FBFCA). Prior to the study, all children in the FBFCA had been initiated on ART; thus, the record review at the FBFCA involved only children on ART. The facilities are both private-not-for-profit but differ in service delivery approaches, including catchment areas and enrolment practices. The study population included all HIV positive infants, children, and adolescents aged 0–18 years, enrolled in both programmes over the study period. Services are generally free of charge under both models.

### 2.2. Description of Health Care Models

The FBFCA of MU-JHU Collaboration was established in 2003 with funding from Columbia University. Its catchment area includes Kampala and Wakiso districts and covers approximately 20 km radius from Mulago hospital in Kampala. Enrolment into the FBFCA occurred between 2003 and 2005 and targeted all HIV-infected family members as a unit. However, HIV-infected pregnant women in PMTCT served as the starting point for identifying other infected family members such as infants, children, and spouses or partners to be enrolled into care. Eligibility of the women included being pregnant, testing HIV positive, attendance of PMTCT clinic, disclosure of HIV status to spouse or partner, willingness to be home visited, and living within 20 km radius from Mulago Hospital. The FBFCA offered comprehensive HIV care, including early infant diagnosis (EID), treatment and psychosocial support services, other medical services, and routine follow-up to eligible women and their families. All children enrolled in the study had been initiated on ART prior to the study start date, in contrast to the CHBC. The programme has a uniform design that has been implemented in many countries. Although funding ended in December 2011, the families continue to be followed in a family care approach with funding from the US President's Emergency Plan for AIDS Relief (PEPFAR).

The CHBC also started in 2003 and it integrates facility-based care with home-based care using community involvements as important linkages to decentralize HIV services. It has a catchment area covering four districts: Kampala, Wakiso, Mukono, and Mpigi within 21 km radius from Nsambya hospital. In contrast to the FBFCA, children and adolescents in the CHBC were identified directly for participation. The components of the CHBC, details of enrolment practice, pre-ART care, and ART care packages, as well as patient tracking system, have been described in an earlier work [27]. The CHBC was funded by international donors and partners and indirectly supported by PEPFAR.

Both health care models share some common elements such as providing additional support including nutritional supplements, patient education, and counselling of patients and their caregivers. Additionally, peer support groups for both adults and children have been developed to promote emotional support. Other components of psychosocial support include financial assistance for income generating activities, a music, dance, and drama group, and home visiting to track defaulting patients. Furthermore, the FBFCA trains peer-educators to provide support and help in the clinics.

### 2.3. Clinical and Laboratory Follow-Up

Patients were followed up routinely using similar appointment systems, standard guidelines, and procedures. Generally, children on ART had monthly clinic visits under both models, while visits for pre-ART patients depended on their clinical conditions and varied between one month under the CHBC and 3–6 months under the FBFCA. Initiation of ART was based on the Ugandan National ART guidelines that are adopted from the WHO guidelines [25, 28–30], which have changed over time, especially with the more recent move in 2013 to initiate treatment in all children under 5 years of age, irrespective of clinical or immune status [11]. During visits, patients were evaluated clinically using WHO clinical staging, age, weight, height, ART status, and laboratory investigations like haemoglobin levels and 6-monthly CD4 cell counts to monitor response to therapy. Adherence to clinical appointments was assessed using appointment schedules, while adherence to medication was assessed by caregivers and self-reports in addition to pill counts. Apart from ART, patients in care received universal Cotrimoxazole prophylaxis for opportunistic infections and secondary prophylaxis for cryptococcal meningitis.

### 2.4. Study Outcomes

The main study outcomes were (a) retention in care, (b) deaths among patients on ART, and (c) pre-ART deaths (deaths before ART initiation) among patients in the CHBC programme. Death was ascertained through medical records and verbal autopsies carried out by trained community volunteers and counsellors. Retention was defined as the proportion of patients known to be alive (either by patient record review or by telephone calls or home visits) and in care at the end of the follow-up period. We defined loss to follow-up (LTFU) as 90 days or more (if on ART) and 180 days or more (if not on ART) without contact since the last clinic appointment. Attrition included deaths and LTFU. Known transfers to continue ART or care at other facilities were not considered as attrition.

### 2.5. Statistical Analysis

We analysed factors that determined retention and mortality among children and adolescents enrolled in the two HIV service delivery models described above. We used frequency distributions, medians, and interquartile range (IQR) to describe baseline characteristics and compared these using Chi-square and Wilcoxon Rank-Sum tests, respectively. The baseline characteristics included age groups (at enrolment), gender, CD4 cell counts, CD4 percent, growth responses (weight-for-age and height-for-age *z*-scores), WHO disease stages, ART status, and age at ART initiation. Because of differences in baseline characteristics in the two study groups, all analyses were adjusted for age at ART initiation, CD4 percent, CD4 cell counts, proportions on ART, nutritional status, and WHO clinical staging using Cox regression. In addition, Cox regression was used to determine factors associated with attrition among patients on ART in both models and among patients in the CHBC separately, in unadjusted and adjusted analyses. Kaplan Meier curves with log rank tests were used to describe and compare retention and survival, stratified by model of care as well as by age groups. Data on CD4 cell count and CD4 percent were log transformed because of skewed distribution. Finally, we used Chi-square test to examine the number and proportions of children dying prior to ART initiation in terms of whether they met or did not meet the 2011 US or 2010 WHO guidelines for initiating ART. All statistical testing was two-sided and conducted at the 5% significance level. Data from both programmes were extracted from databases, merged, and analysed with Intercooled STATA software version 12.

Ethical clearance was approved by the MildMay Institutional Review Board and Ethics Committee, and the study was registered by the Uganda National Council for Science and Technology (UNCST, ref. no. HS 1021). The relevant committees waived informed consent. The study was funded by the University of Padua and supported by Casa Accoglienza alla vita padre Angelo and PENTA Foundation.

## 3. Results

### 3.1. Baseline Characteristics

Overall, 1,623 infants, children, and adolescents were included in the analyses, 90.0% (1460/1623) were in the CHBC (Table 1). There were slightly but not significantly more females compared to males. At enrolment, 47.1% in the CHBC and 38.9% in the FBFCA were over 60 months of age (*P* = 0.097). Baseline median CD4 cell counts were 393 cells/mm^3^ in the CHBC versus 727 cells/mm^3^ in FBFCA (*P* < 0.001) and median CD4 percents were 5.8% in CHBC versus 17.0% in FBFCA (*P* < 0.001). By WHO clinical staging, 86.4% and 96.9% were in stages I-II in the CHBC and FBFCA, respectively, versus 13.6% and 3.1% in stages III-IV in the CHBC and FBFCA, respectively (*P* < 0.001). ART was initiated among 30.2% in the CHBC model compared to 100% in the FBFCA (*P* < 0.0001). Median age at ART initiation was 91.0 months for children in the CHBC versus 45.9 months in the FBFCA (*P* < 0.001). In terms of growth response, 37.4% in the CHBC versus 16.9% in the FBFCA had weight-for-age *z*-scores of ≤−2SD (*P* < 0.001), while 55.7% in the CHBC versus 69.7% in the FBFCA had height-for-age *z*-scores of >−2SD (*P* = 0.001).

### 3.2. Retention in Care

**Figure 1:**
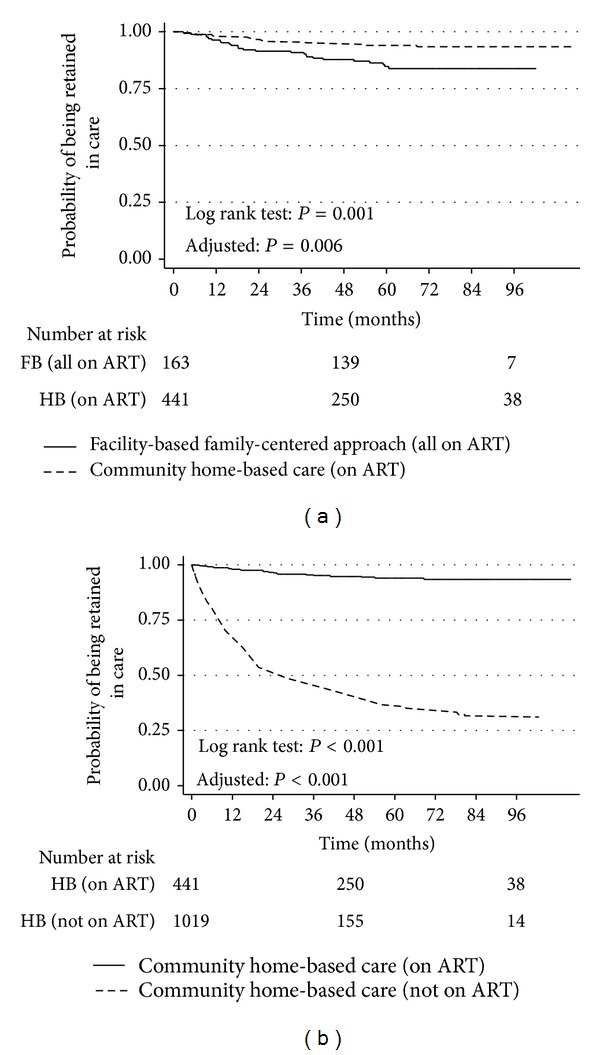
Retention in care, HIV-infected children and adolescents in a facility-based family centred approach (FBFCA) and a community home-based care (CHBC) model, on and not on ART, Kampala, Uganda, 2003–2010. It compares retention among patients on ART in the two models, and it was higher in the CHBC.

An overall average of 4.2 years of follow-up was observed (maximum 7.7 years). Retention in care was substantially and significantly higher among children on ART compared to those not on ART within the CHBC model (*P* < 0.001, before and after adjustment). A total of 266 children were lost to follow-up, all were within the CHBC model (18.2%) with only two (0.5%) on ART lost to follow-up. Among children on ART, retention was higher in the CHBC (94.8%, 95% CI: 92.7%–96.8%) compared to (84.7%, 95% CI: 79.1%–90.2%; *P* = 0.001, adjusted *P* = 0.006) in the FBFCA (Figures 1(a) and 1(b)).

### 3.3. Factors Associated with Attrition among Children and Adolescents on ART in Both Models

In univariate analysis, attrition was significantly associated with model of care (HR: 0.40, 95%CI: 0.23–0.70, *P* = 0.002), mild immunosuppression (HR: 4.17, 95% CI: 1.31–13.31, *P* = 0.016), severe immunosuppression (HR: 3.14, 95% CI: 1.10–8.94, *P* = 0.032), age at ART initiation (HR: 0.74, 95% CI: 0.61–0.90, *P* = 0.003), and weight-for-age *z*-scores of >−2SD (HR: 0.40, 95% CI: 0.21–0.77, *P* = 0.006).

At multivariate modelling, the risk of attrition was significantly associated with model of care (HR: 0.29, 95% CI: 0.12–0.70, *P* = 0.006) with the CHBC model promoting retention, mild immunosuppression (HR: 4.66, 95% CI: 1.21–17.98, *P* = 0.026), and weight-for-age *z*-scores of >−2SD (HR: 0.31, 95% CI: 0.15–0.65, *P* = 0.002, Table 2).

### 3.4. Factors Associated with Attrition among Children and Adolescents in the CHBC (30% on ART)

At univariate analysis, the risk of attrition in the CHBC was significantly associated with age group 36–59 months (HR: 0.46, 95% CI: 0.33–0.64, *P* < 0.001), age group 60+ months (HR: 0.44, 95% CI: 0.35–0.56, *P* < 0.001), CD4 cell count (HR: 0.85, 95% CI: 0.78–0.93, *P* < 0.001), CD4 percent (HR: 0.80, 95% CI: 0.66–0.96, *P* = 0.016), and severe immunosuppression (HR: 1.87, 95% CI: 1.44–2.43, *P* < 0.001). The risk of attrition was also significantly associated with WHO clinical stages III-IV (HR: 1.84, 95% CI: 1.47–2.29,  *P* < 0.001), weight-for-age *z*-scores of >−2SD (HR: 0.76, 95% CI: 0.62–0.93, *P* = 0.007), height-for-age *z*-scores of >−2SD (HR: 0.69, 95% CI: 0.56–0.84, *P* < 0.001), and receipt of ART (HR: 0.06, 95% CI: 0.04–0.09, *P* < 0.001).

At multivariate analysis, the risk of attrition was significantly associated with CD4 cell count (HR: 0.84, 95% CI: 0.74–0.95, *P* = 0.006), WHO clinical stages III-IV (HR: 1.94, 95% CI: 1.34–2.80, *P* < 0.001), and receipt of ART (HR: 0.04, 95% CI: 0.02–0.08, *P* < 0.001, Table 3).

### 3.5. Retention Stratified by Age Groups

**Figure 2:**
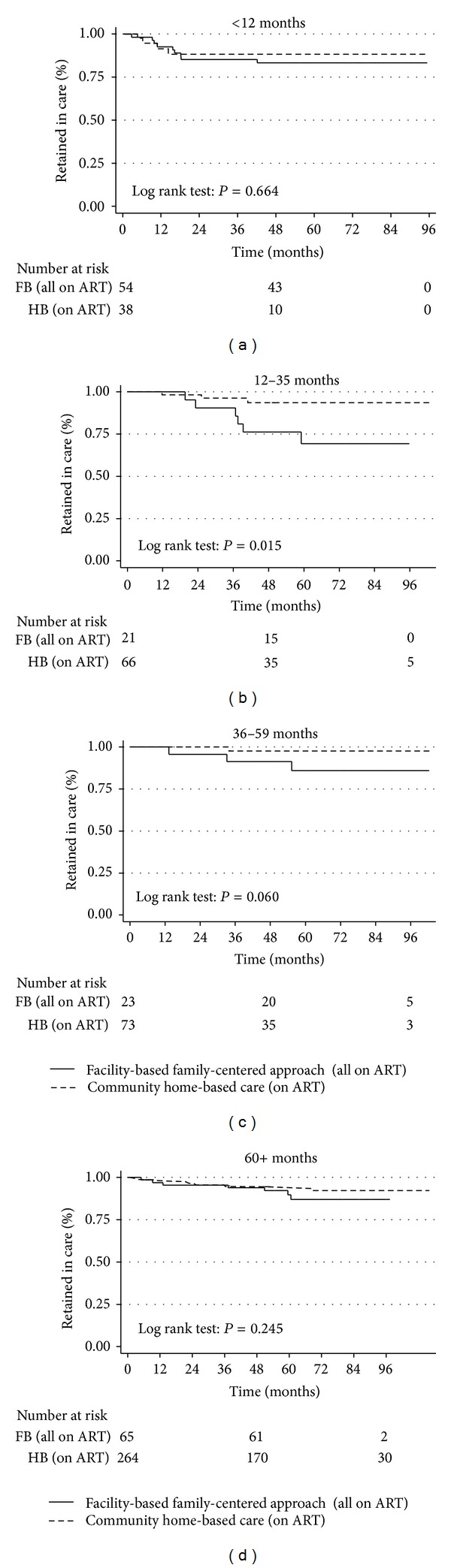
Retention stratified by age group among HIV-infected children and adolescents in a CHBC and a FBFCA in Kampala, Uganda (2003–2010). It stratifies retention by age groups in the two models. Age groups 12–35 months and 36–59 months appear to be confounders for retention.

When retention was stratified by age groups among children and adolescents in the two models, the difference was only significant in the age group 12–35 months (*P* = 0.015) and borderline in age group 36–59 months (*P* = 0.06). This means that age has a confounding effect on retention in care (Figure 2).

### 3.6. Mortality on ART

**Figure 3:**
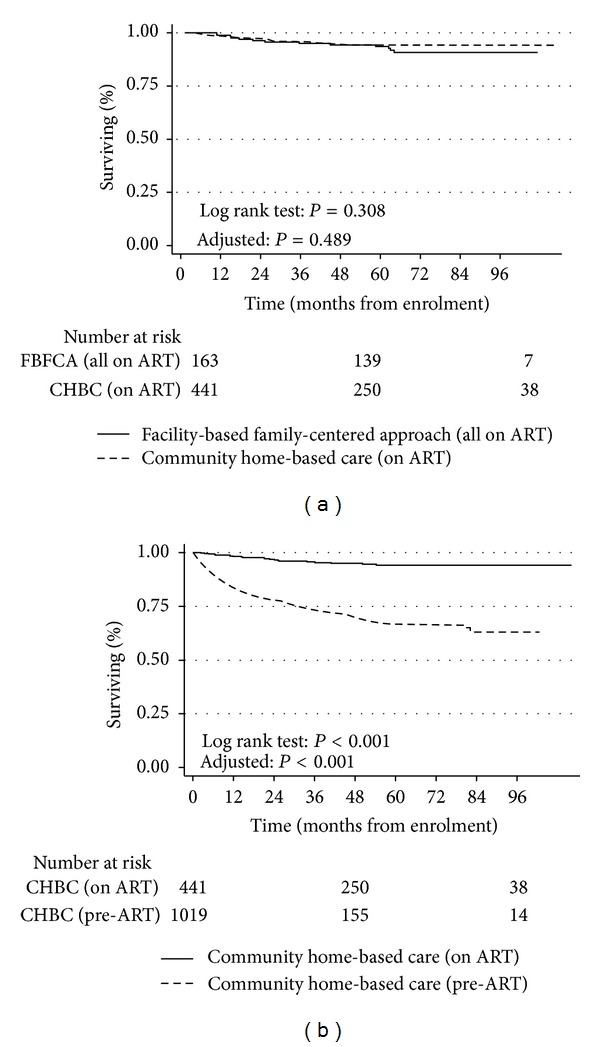
Survival on antiretroviral therapy (ART) among HIV-infected children and adolescents in a facility-based family-centred approach (FBFCA) and a community home-based care (CHBC) model, Kampala, Uganda, 2003–2010. (a) compares survival trends among children and adolescents on ART in both models and shows that they were not significantly different. On the other hand, (b) shows that survival trends differed significantly between patients on ART in the CHBC and those not receiving ART (pre-ART).

There was no significant difference in survival of children and adolescents receiving ART between the two models (Figure 3(a), *P* = 0.308, adjusted *P* = 0.489). However, there was a significant difference between pre-ART deaths (deaths that occurred before ART initiation) and deaths among ART patients in the CHBC (Figure 3(b), log rank test: *P* = 0.001, before and after adjustment). Overall, 34 children died while on ART, 4.8% (21/441) within the CHBC and 8.0% (13/163) within the FBFCA. The estimated time-point mortality for ART patients in the CHBC were 1.9% at 12 months, 5.9% at 60 months, and 5.9% at 96 months. For the FBFCA, estimated time-point mortality at 12, 60, and 96 months were 1.2%, 8.3%, and 9.3%, respectively.

### 3.7. Survival Stratified by Age Groups

**Figure 4:**
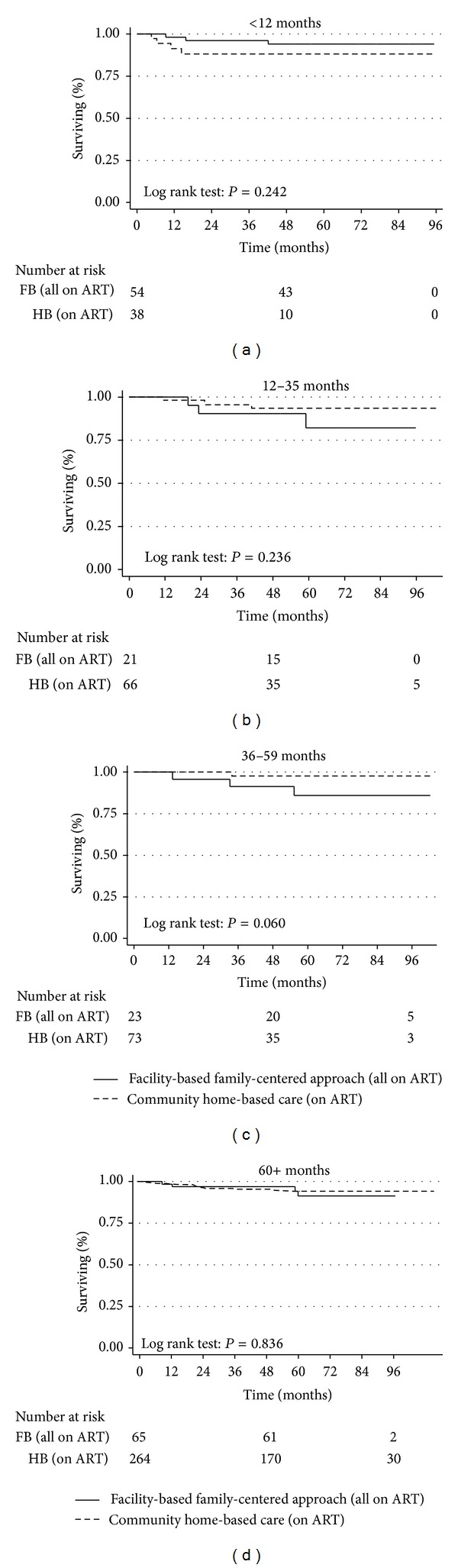
Survival stratified by age group among HIV-infected children and adolescents in a CHBC and a FBFCA in Kampala, Uganda (2003–2010). It stratifies survival by age groups in the two models. Apart from a borderline effect for age group 36–59 months (*P* = 0.060), there was not enough evidence to suggest that age had a confounding effect on survival in the two models.

When survival was stratified by age groups in the two models, there was no significant difference, except a borderline effect for age group 36–59 months (log rank test: *P* = 0.060, Figure 4). Thus, there was not enough evidence to suggest that age had a confounding effect on survival.

### 3.8. Pre-ART Mortality

Sixty deaths were recorded among children in the CHBC who were not on ART (Table 4). Most were known to have met the 2011 US (81.7%) and the 2010 WHO (80.0%) treatment initiation guidelines. All recorded infant deaths (under 12 months) were among those who had met both US and WHO treatment guidelines before they died but had not been initiated on ART related to factors including delayed infant HIV diagnosis, availability of ARVs, and loss to follow-up. In addition, about 20% of deaths occurred in children and adolescents who had not yet met either guideline for initiation.

## 4. Discussion

We found that by far the most significant factor determining retention in care among infants, children, and adolescents with HIV was being on ART. Once on ART, there was no difference in survival between the CHBC and FBFCA. In addition to ART initiation directly promoting survival [31–33], we observed a substantial indirect survival effect by ART dramatically enhancing retention (Figure 1(b)). The CHBC model therefore stands to improve child survival greatly if ART is initiated early. Furthermore, patients can benefit from ancillary interventions to promote health and well-being such as food supplements, adherence counselling, and psychosocial support also effective only if retained in the programmes [34–36]. Of note, the CHBC and FBFCA models reach two different populations of HIV-infected children (Table 1). The FBFCA engages the children at or nearer to birth, whereas the CHBC attempts to find them in the communities and thus complementing each other. Operational synergies between the two models could result in a wider reach and greater ART coverage among HIV-infected children and adolescents.

We also observed that the majority of the pre-ART deaths occurred in children and adolescents who had met the 2011 US and the 2010 WHO treatment guidelines but did not initiate therapy for various reasons. This finding illustrates the failure of timely initiation of ART, which in turn may be linked to programmes that are not tailored to meet the needs of the infected child and family. For instance, at the time of this study, there were no PMTCT/EID services within the CHBC. That scenario, coupled with delayed HIV diagnosis, drug stock-outs, logistic challenges, and fragile linkages to ART initiation and psychosocial support services may have resulted in considerable LTFU and deaths (Figures 1(b) and 3(b)) between testing and initiating care and treatment [37–40] in the CHBC. There is an urgent need for concerted efforts to ensure timely initiation of ART in children and adolescents in the CHBC. To that effect, simplification of treatment guidelines to universal treatment regardless of disease stage would not only help such efforts but also eliminate missed opportunities to initiate ART, while awaiting eligibility criteria to be met [3].

We also saw that nearly one out of every five deaths occurred in children and adolescents who had not yet met eligibility for initiation according to local guidelines. As shown by the literature, significant clinical events do occur in HIV-infected children and adolescents even before meeting the previous guidelines [19, 20]. Our data therefore support the 2013 WHO consolidated guidelines recommending early ART initiation for children less than five years old regardless of CD4 cell count [41]. However, the new guidelines may not address substantial mortality for children over five. Given the number of deaths we observed in children over five who had not met the guidelines, coupled with substantial LTFU when not on ART, we believe the benefits of early ART outweigh the risk of delayed ART for this older group as well. We therefore endorse extending initiation of ART to all HIV-infected children and adolescents irrespective of their CD4 cell counts as a means to avert child deaths.

Among patients on ART in both models, retention was higher in the CHBC. Factors that may explain this observation include decentralized voluntary counselling and testing and ART refills involving outreach clinics within the communities. In addition, ART patients in the CHBC have a “priority” tracking system within the clinics compared to patients not receiving ART, due to scanty resources [27]. However, the “priority” tracking may not have promoted retention of children not on ART. This disparity calls for an integrated approach towards patients tracking. Although all the children were on ART in the FBFCA and retention was relatively stable, the services were not decentralized. Hence, we believe that geographical access may have been a barrier to retention over time. We also think that socioeconomic factors and education level of caretakers may have contributed to the differences in retention.

We also noted that age was a confounder for retention but not survival (Figures 2 and 4). Indeed, when retention was stratified by age groups, more children in the CHBC than in the FBFCA were retained in the age groups 12–35 months and 36–59 months. Many of the children in the FBFCA in those age groups were “graduates” of the PMTCT programme and were exposed to single dose Nevirapine (sd NVP) for PMTCT and were on NVP-based first line ART regimens. Studies from Uganda and elsewhere have shown that exposure to sd NVP during PMTCT and initiating NVP-based first line ART regimens in HIV-infected children was associated with suboptimal clinical outcomes [42–45] and may partly explain this observation. Additionally, maternal health issues could be contributory factors. On the contrary, the CHBC had no PMTCT programme and most probably had more non-NVP-exposed children for those age groups and thus better outcomes on ART. For the age group 5 years plus, retention was not different between the two models, as they were either far removed from exposure or were never exposed to sd NVP.

Additionally, we found that among patients receiving ART, attrition was significantly associated with model of care, mild immunosuppression, and being more underweight (Table 2), whereas in the CHBC, the risk of attrition was significantly associated with CD4 cell count, WHO clinical stages III-IV, and absence of ART (Table 3). These findings have been described consistently in the literature [10, 13, 27].

In terms of growth responses, we saw that children in the CHBC were significantly more underweight, whereas those in the FBFCA were significantly more stunted with some overlap. Although it is well recognized that HIV infection in children compromises growth responses, in Uganda, 39% and 16% of all children less than 5 years are stunted and underweight, respectively, demonstrating that important non-HIV contributors to stunting and wasting exist in particular high background rates of TB and other coinfections, food insecurity, and malnutrition [46, 47]. Children in the FBFCA were relatively younger, mainly enrolled through PMTCT/EID programmes and were all on ART. Thus, the observed growth responses could be related to complications of perinatal HIV infection, younger age, and background factors. In contrast, children in the CHBC were older and only 30% were on ART. In an earlier study [27], we noted that about 46% of the patients in the CHBC were orphans and the majority were malnourished, older, and less likely to receive ART. We therefore think that these factors could be contributing to the observed growth responses in that cohort.

We note potential limitations to our data and conclusions. First, the data were observational and based on programmatic information. Second, the two programmes should be interpreted in the context of reaching different populations of children and the direct comparisons are cautious. Patients were not randomized for one or the other model, but rather circumstances (such as distance to facilities) dictated what options were available. Third, some aspects of the interventions were shared by both models, such as guidelines for initiating ART and nutritional support and could be potentially confounding. Fourth, the high level of loss to follow-up among patients not on ART in the CHBC leaves much doubt on the final disposition of the children in that programme. We expect that many of the children lost to care may have died; however, the characteristics of those who died and those who may have accessed care elsewhere are not known [48–50]. Additionally, there were differences in the study population sizes, proportions in age groups, and baseline characteristics, all of which could be potential confounders. Consequently, the analyses were adjusted, including stratification of retention and survival by model of care and age groups, in order to minimize any potentially confounding effects.

We also acknowledge that our findings reflect data from programmes in one urban setting in East Africa and therefore may not be generalizable to family-centred approach and community home-based care models in other settings. Despite the limitations, we firmly believe that our findings remain valid and relevant.

## 5. Conclusion

We conclude that, irrespective of model of care, children receiving ART had better retention in care and therefore long-term survival. Encouragingly, if children were on ART, then their survival was as good, if not slightly better, in the CHBC compared to the FBFCA. Based on our observations, substantial improvement in child survival can be achieved in either a community-based or a family-care model as long as HIV-infected children are identified early and begun on ART. To ensure this occurs, early identification of HIV-infected children requires strong linkages of pregnant HIV-infected women to PMTCT services and active tracking to ensure all HIV exposed infants receive Polymerase Chain Reaction-based early infant diagnosis. Additionally, rapid early initiation of ART among HIV-infected infants and children is essential. We anticipate the move to early initiation of ART in all HIV-infected children and adolescents in resource-limited settings, irrespective of their CD4 cell counts, will improve survival.

Among ART patients in both models, attrition was significantly associated with model of care, mild immunosuppression, and being underweight. In the CHBC, attrition was significantly associated with CD4 cell count, WHO clinical stages III-IV, and absence of ART.

## Figures and Tables

**Table 1:** Baseline characteristics of HIV-infected children and adolescents in a community home-based care model and a facility-based family-centred approach, Kampala, Uganda, 2003–2010.

Characteristics	Community home-based care approach *N* = 1,460	Facility-based family-centred approach *N* = 163	Total *N* = 1,623	*P* values
	*n* (%)	*n* (%)	*n* (%)	
Age (months)				
<12	371 (25.4)	54 (33.1)	425 (26.2)	0.097
12–35	228 (15.6)	21 (12.9)	249 (15.3)	
36–59	173 (11.9)	23 (14.1)	196 (12.1)	
60+	688 (47.1)	65 (38.9)	753 (46.4)	
Gender				
Female	746 (51.1)	87 (53.4)	833 (51.3)	0.581
Male	714 (48.9)	77 (46.6)	790 (48.7)	
Cd4 count and percent				
Median CD4 (IQR)	393 (51–762)	727 (442–1348)	438 (78–824)	<0.001
Median CD4% (IQR)	5.8 (0.1–16.9)	17.0 (10.0–25.0)	8.1 (0.2–18.1)	<0.001
Level of immunosuppression by CD4				
Not significant	273 (24.4)	39 (25.0)	312 (24.4)	0.424
Mild	122 (10.8)	21 (13.5)	143 (11.1)	
Advanced	131 (11.6)	12 (7.7)	143 (11.1)	
Severe	605 (53.5)	84 (53.8)	689 (53.5)	
On ART				
Yes	441 (30.2)	163 (100)	604 (37.2)	<0.001
No	1019 (69.8)	0 (0.0)	1019 (62.8)	
Age at ART initiation (months)			
Median (IQR)	91.0 (48.9–135.5)	45.9 (6.5–85.0)	78.6 (34.0–124.6)	<0.001
WHO clinical staging				
I-II	1258 (86.4)	157 (96.9)	1415 (87.5)	<0.001
III-IV	198 (13.6)	5 (3.1)	203 (12.5)	
Growth response weight-for-age				
≤−2SD	430 (37.4)	24 (16.9)	454 (35.2)	<0.001
>−2SD	719 (62.6)	118 (83.1)	837 (64.8)	
Height-for-age				
≤−2SD	508 (44.3)	43 (30.3)	551 (42.8)	0.001
>−2SD	639 (55.7)	99 (69.7)	738 (57.2)	

**Table 2:** Factors associated with *attrition* among HIV-infected children and adolescents *on ART* in a community home-based care model and a facility-based family-centred approach, Kampala, Uganda, (2003–2010) using Cox regression.

Characteristics	Unadjusted (univariate analysis)		Adjusted* (multivariate analysis)	
	HR^‡^ (95% CI)	*P*	HR^‡^ (95% CI)	*P*
Model of care				
FBFCA	1	0.002	1	0.006
CHBC	0.40 (0.23–0.70)		0.29 (0.12–0.70)	
Age (months)				
<12	1		1	
12–35	0.73 (0.31–0.70)	0.465	1.95 (0.39–9.89)	0.419
36–59	0.29 (0.09–0.87)	0.028	1.63 (0.18–15.17)	0.664
60+	0.43 (0.22–0.85)	0.015	3.19 (0.27–37.03)	0.354
Gender				
Female	1	0.675		
Male	1.13 (0.64–1.99)			
Cd4 count and percent				
CD4^†^	0.86 (0.69–1.06)	0.162		
CD4%^†^	1.00 (0.57–1.77)	0.992		
Level of immunosuppression by CD4				
Not significant	1		1	
Mild	4.17 (1.31–13.31)	0.016	4.66 (1.21–17.98)	0.026
Advanced	0.87 (0.16–4.73)	0.868	0.89 (0.09–9.12)	0.921
Severe	3.14 (1.10–8.94)	0.032	3.16 (0.81–12.39)	0.099
Age at ART initiation				
Months^†^	0.74 (0.61–0.90)	0.003	0.67 (0.31–1.44)	0.309
WHO clinical staging				
I-II	1	0.309		
III-IV	1.52 (0.68–3.38)			
Growth response Weight-for-age				
≤−2SD	1	0.006	1	0.002
>−2SD	0.40 (0.21–0.77)		0.31 (0.15–0.65)	
Height-for-age				
≤−2SD	1	0.060		
>−2SD	0.54 (0.28–1.03)			

^‡^Hazard of attrition; ^†^log transformed due to skewed data;

*only factors significant at univariate or borderline were considered into multivariate.

**Table 3:** Factors associated with *attrition* among HIV-infected children and adolescents in the CHBC model, Kampala, Uganda (2003–2010) using Cox regression.

Characteristics	Unadjusted (univariate analysis)		Adjusted* (multivariate analysis)	
	HR^‡^ (95% CI)	*P*	HR^‡^ (95% CI)	*P*
ART				
No	1	<0.001	1	<0.001
Yes	0.06 (0.04–0.09)		0.04 (0.02–0.08)	
Age (months)				
<12	1		1	
12–35	0.85 (0.65–1.10)	0.218	2.18 (0.97–4.92)	0.060
36–59	0.46 (0.33–0.64)	<0.001	1.65 (0.74–3.69)	0.225
60+	0.44 (0.35–0.56)	<0.001	1.38 (0.63–3.01)	0.418
Gender				
Female	1	0.243		
Male	1.11 (0.93–1.33)			
Cd4 count and percent				
CD4^†^	0.85 (0.78–0.93)	<0.001	0.84 (0.74–0.95)	0.006
CD4%^†^	0.80 (0.66–0.96)	0.016		
Level of immunosuppression by CD4				
Not significant	1			
Mild	0.99 (0.66–1.49)	0.968		
Advanced	0.89 (0.59–1.34)	0.590		
Severe	1.87 (1.44–2.43)	<0.001		
Age at ART initiation				
Months^†^	0.85 (0.66–1.10)	0.209		
WHO clinical staging				
I-II	1	<0.001	1.94 (1.34–2.80)	<0.001
III-IV	1.84 (1.47–2.29)			
Growth response weight-for-age				
≤−2SD	1	0.007		
>−2SD	0.76 (0.62–0.93)			
Height-for-age				
≤−2SD	1	<0.001	0.81 (0.60–1.09)	0.162
>−2SD	0.69 (0.56–0.84)			

^‡^Hazard of attrition; ^†^Log transformed due to skewed data;

*only factors significant at univariate or borderline were considered into multivariate.

**Table 4:** Deaths among children and adolescents in community home-based care who were not on ART classified according to whether the US 2011 or WHO 2010 initiation guidelines were met or not, Kampala, Uganda, 2003–2010.

Age group for US (months)	*N*	US CDC guidelines^1^ for ART met	Age group for WHO (months)	*N*	WHO guidelines^2^ for ART met
<12	11	11 (100%)	<12	11	11 (100%)
12–35	13	10 (76.9%)	12–24	9	9 (100%)
36–59	4	3 (75.0%)	24–59	8	5 (62.5%)
60+	32	25 (78.1%)	>60	32	23 (71.9%)

Overall	60	49 (81.7%)	Overall	60	48 (80.0%)

Source: WHO and US treatment guidelines.

^1^US CDC 2011 criteria for ART initiation: <12 months all should be on ART; 12–35 months if CD4 < 1000 or <25%; 36–59 months if CD4 < 750 or <25%; 60+ months if CD4 < 500.

^2^WHO 2010 criteria for ART initiation: <12 months all should be on ART; 12–24 months all should be ART; 24–59 months if CD4 < 750 or <25%; 60+ months if CD4 < 350.
